# Can Remote Monitoring Measure Life Activity and Caregiver Experience? Early Results of Multi-modal Assessments

**DOI:** 10.1093/geroni/igab046.3570

**Published:** 2021-12-17

**Authors:** Walter Dawson, Allison Lindauer, Sarah Gothard, Leslie Tran, Zachary Beattie, Jeffrey Kaye

**Affiliations:** 1 Oregon Health & Science University and Global Brain Health Insitute, Portland, Oregon, United States; 2 Oregon Health Sciences University, Portland, Oregon, United States; 3 Oregon Health & Science University, Portland, Oregon, United States

## Abstract

Subjective assessments of dementia caregiver burden are vulnerable to recall and recency biases. Objective continuous home assessment using passive technologies (e.g., bed mats, actigraphy watches) can provide ecologically valid detail on caregiver stress and family function. We tested the utility of objective assessment of activity before, during and after the behavioral intervention of STELLA (Support via Technology: Living and Learning with Advancing AD) which facilitates effective online management of behavioral symptoms of dementia. We present preliminary data on objective measures of sleep and step counts, and subjective measures of burden. We captured data from three caregivers caring for a family member with dementia. Each family lives in home with unobtrusive monitoring devices that recorded data on sleep (Emfit sleep mat) and daily steps (Withings watch). Self-report assessments of burden, depression and grief were collected prior to and after the 2-month intervention. Objective data was collected continuously. Pre/post subjective assessments suggest that the STELLA intervention has the potential to reduce behavioral symptom frequency and caregiver reactivity to symptoms (pre-STELLA behavior frequency=44.9, post=39.2; pre-STELLA reactivity=50.5; post=38.5). Step count ranged from 775 steps/day to 5065, with each participant trending fewer steps during the intervention. Mean sleep time ranged from 6.3 to 8.6 hours and didn’t change during the intervention. The small sample size limits interpretation but provides evidence that it is feasible to collect continuous objective life-activity data during caregiver interventions. This digital data has the potential to inform the validity of subjective findings by limiting recall and recency biases.

